# The Western diet: a blind spot of eating disorder research?—a narrative review and recommendations for treatment and research

**DOI:** 10.1093/nutrit/nuz089

**Published:** 2019-12-17

**Authors:** Agnes Ayton, Ali Ibrahim

**Affiliations:** 1 University of Oxford, Oxford, United Kingdom; 2 South London and Maudsley NHS Foundation Trust, Snowsfields Adolescent Unit, Mapother House, Maudsley Hospital, London

**Keywords:** anorexia nervosa, binge eating disorder, bulimia nervosa, diet, metabolism, neurobiology

## Abstract

Over the last 50 years, in parallel with the obesity epidemic, the prevalence of eating disorders has increased and presentations have changed. In this narrative review, we consider recent research exploring the implications of changing patterns of food consumption on metabolic and neurobiological pathways, a hitherto neglected area in eating disorder research. One of the major changes over this time has been the introduction of ultra-processed (NOVA-4) foods, which are gradually replacing unprocessed and minimally processed foods. This has resulted in the increased intake of various sugars and food additives worldwide, which has important metabolic consequences: triggering insulin and glucose response, stimulating appetite, and affecting multiple endocrine and neurobiological pathways, as well as the microbiome. A paradigm shift is needed in the conceptual framework by which the vulnerability to, and maintenance of, different eating disorders may be understood, by integrating recent knowledge of the individual metabolic responses to modern highly processed foods into existing psychological models. This could stimulate research and improve treatment outcomes.

## INTRODUCTION

Eating disorders include anorexia nervosa (AN), bulimia nervosa (BN), binge eating disorder (BED), and their variants. They usually start in adolescence, but often continue to adulthood, and are associated with a high rate of multimorbidity^1–3^ and increased mortality.^4^ According to recent studies, eating disorders affect 2%–5% of the population in Western countries,^5–7^ and there are reports of increasing rates worldwide.^8^ Current evidence–based psychological treatments focus on individual or family maintaining factors.^9^^,^^10^ Of these, family therapy has been shown to be the most effective for adolescents,^11^ and cognitive behavioral therapy for adults.^10^ Both of these approaches are recommended across the diagnostic spectrum in line with Fairburn et al’s transdiagnostic model,^12^^,^^13^ which suggests that all eating disorders share the basic psychopathology of overevaluation of weight and shape and their control^13^ and that addressing these issues is an essential component of treatment. Alternative psychological treatment options are also available for adults with AN,^14–16^ but there is no clear difference in outcomes.

The underlying biological factors that contribute to the development of eating disorders remain poorly understood.^17^ Recent advances in the understanding of these factors include those within the areas of neurobiology, immunology, and genetics.^18–22^ A growing body of evidence suggests involvement of metabolic processes in the development of AN, including appetite-satiety pathways. For example, 2 large genome-wide association studies of AN have found not just positive genetic correlations with other mental disorders but also with metabolic parameters, such as high-density lipoprotein cholesterol and significant negative genetic correlations with body mass index, insulin, glucose, and lipid phenotypes.^23^^,^^24^

These findings highlighted, for the first time, the importance of potential underlying metabolic factors, which up until this point have been unexplored. These discoveries can generate new hypotheses, with the potential for reshaping current understanding of the etiological factors involved in eating disorders and improving treatment. This is particularly important because despite decades of treatment research, only 30%–40% of patients achieve full remission with current psychological treatments.^25–27^

To date, very little research has been conducted on the impact of the modern Western diet on abnormal eating behaviors and related eating disorder psychopathology. This narrative review explores how modern dietary patterns and their metabolic consequences contribute to the development and maintenance of eating disorders.

## CHANGING PATTERNS OF EATING DISORDERS OVER TIME

In contrast with other mental disorders, historical descriptions of eating disorders have been rare, and the presentations have significantly changed over time. The first medical record of a patient engaging in self-starvation for psychological reasons was made in 1689 in England. The term *anorexia nervosa* was introduced almost 200 years later, in 1874, but it was still a very rare and therefore little-studied condition.^28^ In the 1960s, the morbid fear of fatness was described as a core feature of the disorder, which was regarded as a culture-bound syndrome affecting mainly young women of higher social classes in Western countries.^29^^,^^30^

A dramatic 400% increase in incidence was noted in the United States between the 1960s and 1970s, but the incidence rate was still around 5 cases per 100 000 individuals.^31^ Thirty years later, a UK study showed a further increase in the incidence rate for AN – to approximately 20 cases per 100 000.^32^ Recent epidemiological studies estimate 0.4%–1% prevalence rates in Western countries.^6^^,^^33^ Incidence of AN is lower in the Far East than in Western countries, but trends are increasing.^8^^,^^34–36^

Bulimia nervosa emerged much more recently: the term was first introduced in 1979,^37^ and the diagnosis was added to the *Diagnostic and Statistical Manual of Mental Disorders* (DSM) in 1980.^38^ The rate of BN has been increasing, not just in Western countries but worldwide, calling the culture-bound theory into question. Current prevalence rates are around 1%–2% in different studies.^35^^,^^36^^,^^39^^,^^40^

Later still, into the mid-1980s, binge eating disorder became recognized as a distinct entity, and was finally introduced as a new diagnostic category in 2013 in the fifth edition of the DSM. Current epidemiological data suggest that this is the most common form of eating disorder, affecting 2%–3% of the population in Western countries.^41^^,^^42^

One can ask what factors changed within society over this time period to account for the increasing prevalence and changing presentation of eating disorders. Historically, it was hypothesized that eating disorders develop in societies that value thinness. The culture-bound hypothesis attributed this to the use of images of thin women in the media in Western countries.^43^ This was very influential, but has been called into question with the observation of increasing worldwide trends in eating disorders in parallel with industrialization and urbanization.^36^ One of the major changes over the last few decades has been the increasing domination of industrial food processing, which has led to the replacement of traditional eating patterns.^44^

## EPIDEMIOLOGICAL LINK BETWEEN THE OBESITY EPIDEMIC AND EATING DISORDERS

The obesity epidemic started in the 1970s, initially in the United States and subsequently spreading worldwide.^45^ Approximately 60%–80% of adults, and 20%–30% of children in most Western countries, are overweight or obese, which is unprecedented in human history.^46–48^ In parallel, there has been an increase in the incidence of metabolic diseases, such as diabetes and nonalcoholic fatty liver disease, not just in adults but also in children.^49^ Currently, the global prevalence of diabetes is 8.8%, and that for prediabetes is much higher.^50^

Surprisingly, the causes of the parallel increase in obesity and metabolic and eating disorders are rarely examined together. This is maintained by current research funding allocations: research funding for eating disorders is 3% of that spent on obesity in the United States (https://report.nih.gov/categorical_spending.aspx), and the situation is similar elsewhere.^51^ A recent large population study showed that although genetic predisposition interacts with the obesogenic environment, since the 1980s, body mass index has increased for both genetically predisposed and non-predisposed individuals, implying that the environment remains the main contributor to the obesity epidemic.^52^ Genetic research into the etiology of eating disorders is in its infancy, but the increasing prevalence of BN and BED worldwide suggests that the same environmental factors may be at play.^52^

**Figure 1:**
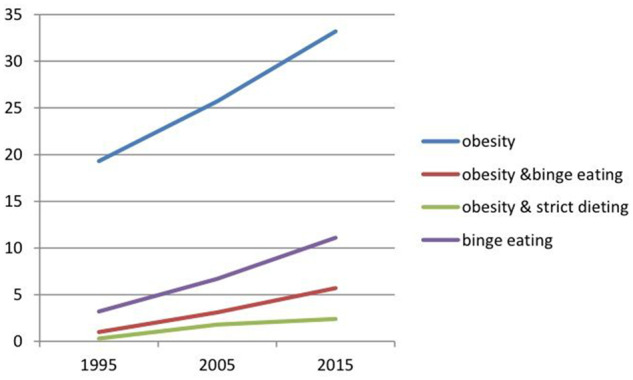
**Prevalence of obesity and comorbid eating disorder behaviors in South Australia from 1995 to 2015^53^**

A recent population-based Australian study, focusing on the trends between 1995 and 2015, showed significant increases in the prevalence of both obesity (19%–33%) and binge eating (3%–11%), with the highest increases in the prevalence of obesity with comorbid binge eating (7.3-fold) or obesity with comorbid, very strict dieting/fasting (11.5-fold) (Figure 1).^53^

Furthermore, the association between diabetes and eating disorders, particularly BN and BED, is being increasingly recognized.^54^^,^^55^ A recent systematic review and meta-analysis found that the risk of type 2 diabetes is approximately 3 times higher among those with BN and BED, whilst the risk of developing type 2 diabetes is reduced in those with AN.^56^ These findings provide strong support for the possibility that disturbed carbohydrate metabolism may contribute to the onset and maintenance of BED and BN. This could be due to underlying hyperinsulinemia-related hunger and satiety dysregulation, which may pre-exist both conditions and is worsened by high-carbohydrate food choices.^57^ The possible mechanism will be discussed later.

It is well demonstrated that eating disorders are more common in young women, but there has been an increased incidence of such disorders in both males and middle-aged people.^5^^,^^58^ The obesity epidemic is associated with increased rate of dieting, which is a well-known risk factor for the development of all eating disorders.^38^^,^^59^ The emergence of the weight-loss industry actively encourages abnormal eating behaviors, including food restriction and calorie counting and the promotion of highly processed “diet products.”

The current psychological models do not explore the ultimate causes of dieting and abnormal eating behaviors in the context of an obesogenic environment. Dieting has become common in Western societies: a recent large population-based study in Norway found that 58.8% of women were dissatisfied with their weight, and 54.1% of the women reported dieting.^60^ Another large population-based study in the Netherlands found that dieting and fear of weight gain are common for women throughout their entire life span, though this is also the case for a substantial number of men. Dieting was most frequently reported by both sexes in middle age. For men, fear of weight gain was related to increasing weight in middle age, whilst fear of weight gain was most prevalent in women aged between 16 and 25 years (73.2%–74.3%), even though this age group is the least likely to be overweight. This mirrors the onset of eating disorders. Notably, approximately 10 times as many women (12.5%) than men (1.5%) reported extreme fear of gaining weight, which is consistent with gender differences in eating disorders.^61^ These findings are compatible with Abed’s intrasexual competition hypothesis of eating disorders.^62^ He argued that the high risk of obesity in modern societies and the delay of reproductive age together reinforce the need for weight and shape control to improve or maintain physical attractiveness in “mate attraction” and “mate retention” strategies. One can argue that dieting in an obesogenic environment is an adaptive strategy for women approaching reproductive age to maximize their attractiveness.^63^^,^^64^ The increasing rates of “muscularity-oriented eating disorders” in men are consistent with this hypothesis, as strength and fitness are important sexual selection pressures for males.^65^^,^^66^ However, although dieting is very common, additional vulnerability factors are necessary for the development of eating disorders. One recent genetic study found that metabolic factors, including glucose and lipid metabolism, play an important role in the etiology of AN.^24^

## THE CHANGING FOOD ENVIRONMENT OVER THE LAST 50 YEARS

**Figure 2:**
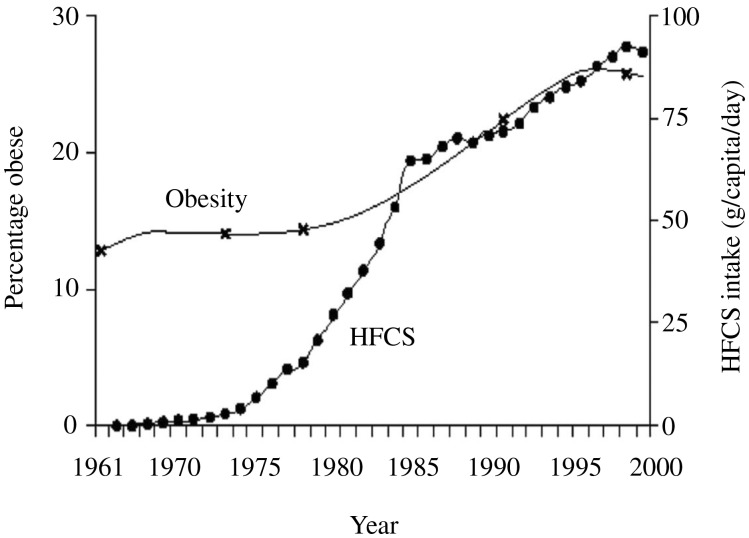
**Association between high-fructose corn syrup and obesity in the United States.^69^** *Abbreviation:* HFCS, high-fructose corn syrup

The most notable change over the last 50 years has been a shift in food consumption and dietary patterns within the population as a whole. A number of factors have simultaneously contributed to this. The first US dietary guidelines in 1977 set the scene by encouraging the consumption of 50%–60% of calories from carbohydrates and recommending a reduction in saturated fats, replacing them with vegetable oils.^67^ This was further reinforced by the food industry promoting “healthy” low-fat products, with their higher proportion of added sugars, additives, emulsifiers, and trans fats. Acceleration in food science techniques in the 1980s enabled the invention of a new range of palatable products with a long shelf life and made from cheap ingredients and additives. For example, high-fructose corn syrup was invented in North America in 1975. It is consumed at a rate of 27.5 kg per capita in the United States^68^ and has been spreading worldwide as the market is increasingly controlled by a small number of multinational companies. This parallels the increasing prevalence of obesity (Figure 2).^69^

Sugar in its various forms, as well as carbohydrates from cereals or corn, is less expensive than protein or fats, and this has favored the incorporation of such foods into processed food products that maximize profits^70^ and have been heavily promoted as healthier alternatives to traditional foods. This has led to the mass consumption of ultra-processed foods that have been stripped of their naturally occurring nutrients, which have been replaced by food additives. The NOVA classification system has recently been proposed as a framework for categorizing the degree of industrial food processing (Table 1).^71^ Although it has been criticized by industry representatives,^72^ as it is not concerned with macronutrient content, it is nevertheless an important framework for assessing levels of industrial food processing. In general, ultra-processed foods are high in sugars and fats and low in natural protein.^73^^,^^74^ Studies based on NOVA show exponential growth in consumption of ultra-processed products and confirm that such products have gradually been displacing unprocessed or minimally processed foods and freshly prepared dishes and meals. Moreover, ecological and epidemiological studies have shown a link with increased rates of obesity and metabolic disorders.^71^^,^^75^^,^^76^ Research suggests that in high-income countries, more than half of the foods consumed are ultra-processed for most age groups, including children.^77^^,^^78^ This may even be an underestimate, as 75% of supermarket foods include added sugars, which is a marker of ultra-processed foods.^79^ Less developed countries are rapidly catching up, as food supplies are now becoming part of a global food system increasingly dominated by ready-to-consume processed products and enabled by international trade agreements.^80^^,^^81^

**Table 1:** NOVA classification of food processing^70^

NOVA category	Description	Examples
NOVA 1	**Unprocessed** (or natural) foods are edible parts of plants or of animals (muscle, offal, eggs, milk), and also fungi, algae. **Minimally processed** foods are natural foods altered by processes that include removal of inedible or unwanted parts, and drying, crushing, grinding, fractioning, filtering, roasting, boiling, nonalcoholic fermentation, pasteurization, refrigeration, chilling, freezing, placing in containers, and vacuum-packaging. These processes are designed to preserve natural foods, to make them suitable for storage. Many unprocessed or minimally processed foods are prepared and cooked at home.	Fresh or frozen fruit vegetables, eggs, meat, fish, algae Natural yogurt, sour cabbage, kimchi Home-cooked meals with natural ingredients
NOVA 2	**Processed culinary ingredients** are substances derived from Group 1 foods or from nature by processes that include pressing, refining, grinding, milling, and drying. They are not meant to be consumed by themselves, and are normally used in combination with Group 1 foods to make freshly prepared drinks, dishes, and meals.	Flour, olive oil, butter, coconut oil, cream, sour cream, dried fruit and vegetables, herbs Ingredients in home cooking
NOVA 3	**Processed foods**, such as bottled vegetables, canned fish, fruits in syrup, cheeses, and freshly made breads, are made essentially by adding salt, oil, sugar, or other substances from Group 2 to Group 1 foods. Processes include various preservation or cooking methods and, in the case of breads and cheese, nonalcoholic fermentation. Most processed foods have 2 or 3 ingredients and are recognizable as modified versions of Group 1 foods.	Canned fish, cheeses, freshly made bread, canned vegetables
NOVA 4	**Ultra-processed foods**, such as soft drinks, sweet or savory packaged snacks, reconstituted meat products, and preprepared frozen dishes, are not modified foods but formulations made mostly or entirely from substances derived from foods and additives, with little if any intact Group 1 food. Ingredients of these formulations usually include those also used in processed foods, such as sugars, oils, fats, or salt. But ultra-processed products also include other sources of energy and nutrients not normally used in culinary preparations. Some of these are directly extracted from foods, such as casein, lactose, whey, and gluten. Many are derived from further processing of food constituents, such as hydrogenated or interesterified oils, hydrolyzed proteins, soya protein isolate, maltodextrin, invert sugar, and high-fructose corn syrup. Classes of additives found only in ultra-processed products include dyes and other colors, and color stabilizers; flavors, flavor enhancers, and non-sugar sweeteners; and processing aids such as carbonating, firming, bulking and anti-bulking, de-foaming, anti-caking, and glazing agents, emulsifiers, sequestrants, and humectants. A multitude of sequences of processes is used to combine the usually many ingredients and to create the final product (hence *ultra-processed*). The processes involved include several with no domestic equivalents, such as hydrogenation and hydrolyzation, extrusion and molding, and preprocessing for frying.	Soft drinks Sweet and savory packaged snacks Diet and low-fat products Products with artificial sweeteners Ready meals Reconstituted meat and fish products Oven chips Margarines Protein bars and drinks

These shifts in the food environment have resulted in dramatic increases in obesity and related chronic noncommunicable diseases, most notably diabetes and metabolic syndrome—at first in high- and middle-income countries, and now also in lower-income ones.^44^^,^^47^ NOVA-4 food consumption is higher in lower socioeconomic groups,^78^^,^^82^ resulting in reduced dietary protein density and increased sugar intake,^83^^,^^84^ which in turn is linked with higher rates of obesity and metabolic^85^ and binge eating disorders.^86^

Fiji provides an interesting example of separate observations of increasing obesity and metabolic and eating disorders after having introduced ultra-processed foods within a short period of time. Becker et al.^87^^,^^88^ recorded an increasing rate of BN following the introduction of US culture. Their inquiry focused on social pressures, such as the popularity of thin characters presented in television dramas. However, this line of investigation did not take into account that Westernization also had a major impact on food consumption patterns and the food environment.^80^ At the same time, there was an introduction and heavy marketing of American foods in supermarkets and television adverts, resulting in a major shift in food consumption away from traditional foods, such as fish and seasonal fruit and vegetables, to highly processed foods, and a consequent rapid increase in obesity, diabetes, and metabolic disorders.^89^^,^^90^ Interestingly, Fijians have a significantly higher insulin response to fructose than people of European origin, so they may have been particularly vulnerable to being affected by this change in diet.^91^

**Figure 3:**
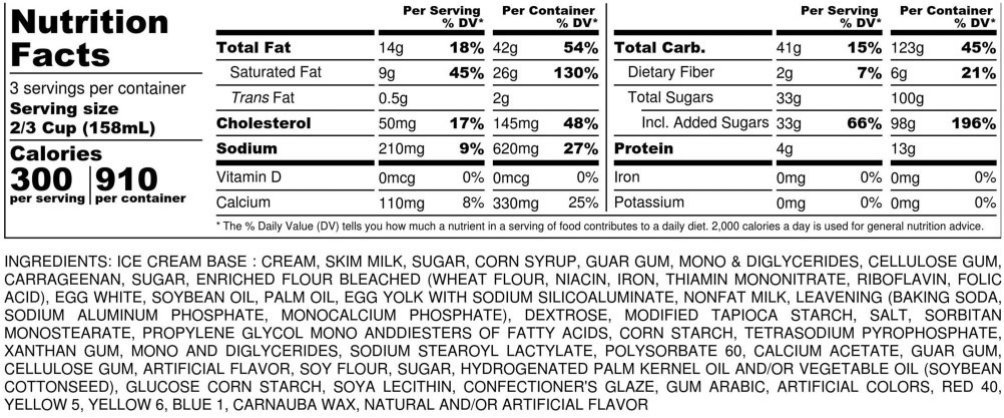
An example of ingredients of a typical NOVA-4 binge food: ice cream

**Figure 4:**
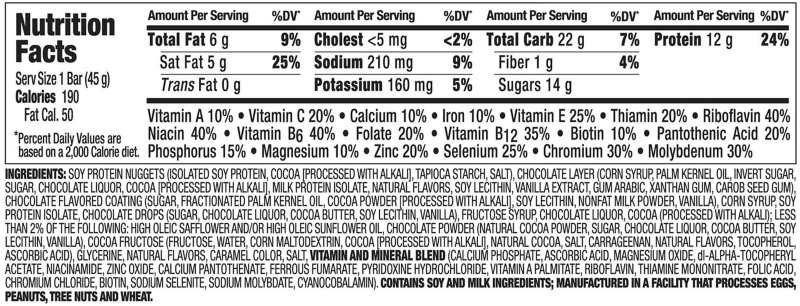
An example of the ingredients of a typical diet food: protein bar

Overlooking the dietary factors contributing to the development of eating disorders in favor of media influences seems to be a consistent blind spot in eating disorder research: social and individual psychological factors are regarded as more important than biological factors, such as metabolic responses to changes in diet.^92^ For example, only a few studies have investigated the dietary habits of people with eating disorders. One recent prospective cohort study of 11 800 Spanish women found that following a Mediterranean diet (involving high consumption of olive oil) is associated with a reduced risk of eating disorder.^93^ Clinical experience suggests that patients do consume a large amount of ultra-processed foods; these include cereals and diet products such as zero-calorie drinks.^94^ This review did not reveal any previous publications exploring the link with eating disorders, and this is urgently needed. Fairburn’s book *Overcoming Binge Eating*^38^ lists typical binge foods, which are almost entirely NOVA-4 foods (Table 2). A case-note study from the present authors' own research population revealed that patients with eating disorders consume 60%–80% ultra-processed foods independent of the diagnostic categories, and 100% of binge foods are classed as NOVA 4 (in press) Examples of binge foods and their ingredients are provided in Figures 3 and 4. Although no studies examining the link between ultra-processed foods and eating disorders were located, NOVA-4 foods did not exist until the 1970s, and their introduction parallels both the rising incidence of obesity and the increased rates of BN and BED.

**Table 2:** List of binge foods reported by patients^38^: macronutrient composition and NOVA categories

	Carbohydrate, %	Fat, %	Protein, %	NOVA category
Ice cream	47	46	7	4
Doughnut	40–45	45–50	2–3	4
Pita bread and hummus	50	39	11	4
Raisin bagels	80	6	8	4
Cookies	60	38	2	4
Nuts	12	75	13	1 or 4
Diet soda	Artificial sweetener	0	0	4
Potato chips	35	60	5	4
Chocolate cake	57	40	3	4
Cherry yoghurt	75	10	15	4
Pizza	48	37	15	4

**Table 3:** Summary and implications for treatment and future research

1. Eating disorder presentations and prevalence have increased over the last 50 years in parallel with the obesity epidemic.
2. The introduction of NOVA-4 ultra-processed foods containing combinations of high-sugar, high-fat and additives not found in nature have transformed food consumption, initially in Western countries and spreading worldwide, gradually replacing natural foods.
3. Ultra-processed foods have been shown in both animal and human experiments to promote overeating, obesity, insulin resistance, and metabolic disorders.
4. The obesogenic environment has resulted in widespread dieting, which is a well-known risk factor for eating disorders.
5. Many features of eating disorders, such as fat phobia, calorie counting, dieting, and exercise, are constantly reinforced not just by the food and weight-loss industries, but also by dietary guidelines, which encourage such reinforcement.
6. The multiple complex metabolic responses to ultra-processed foods are likely to contribute to abnormal eating and the development of eating disorders.
7. The high rate of comorbidity between diabetes and eating disorders suggests shared underlying pathomechanisms relating to impaired carbohydrate metabolism and insulin regulation.
8. High-carbohydrate and processed foods override the brain satiety mechanisms and are, therefore, unlikely to be helpful for patients with tendencies to binge, regardless of the diagnostic categories.
9. Existing treatment models recommend following current dietary guidelines, which continue to advocate low-fat and ultra-processed foods. Considering the effects of these foods on the individual patient would improve engagement and outcomes.
10. Recent research shows that there is wide variation in blood glucose response in the population and that exploration of individual glucose response to food with CGM may be helpful as a psychoeducational tool.
11. There have been no studies exploring the effects of different dietary approaches in the treatment of eating disorders: these are long overdue.
12. An HFLC diet may be beneficial for patients with various eating disorders, particularly for patients with BN and BED and comorbid diabetes. It could improve satiety, metabolic health and body composition, and blood glucose levels. Such a diet may also be beneficial for careful refeeding programs for patients with AN and lead to a reduction of refeeding complications.

*Abbreviations:* AN, anorexia nervosa; BED, binge eating disorder; BN, bulimia nervosa; CGM, continuous glucose monitoring; HFLC, high-fat, low-carbohydrate

## CONSEQUENCES OF THE MODERN FOOD ENVIRONMENT ON APPETITE AND METABOLIC PATHWAYS

Despite intensive research, the causes of the obesity epidemic remain much debated.^47^^,^^52^ The prevailing view has been that this is a question of energy imbalance between input and expenditure. Whilst this may be true, the calories-in, calories-out hypothesis does not explain the reasons for the overconsumption that affects approximately 80% of the population. Overeating is commonly seen as an individual failure in the context of widely available hyperpalatable foods. However, decades of guidelines promoting reduced calorie intake and increased exercise have been ineffective in halting the obesity epidemic,^95^ despite this message being amplified by advertising in supermarkets, on television, in magazines, and also in schools and hospitals. Low-fat and calorie-counted products have become ubiquitous, and are recommended and seen as the healthy option by the population, but they have quite clearly not stopped the obesity epidemic.

The diet and fitness industry has emerged over the last 50 years and has further reinforced the calories-in, calories-out hypothesis. Smartphone applications and wearable technologies provide new reinforcements for calorie counting and exercise monitoring, and indeed many patients with eating disorders use them to control their weight.^96^

The parallels between the dietary guideline recommendations and eating disorder preoccupations and behaviors are striking. These include fat avoidance, calorie counting, and excessive exercise. This raises the question whether dietary guidelines and the calories-in, calories-out hypothesis may have inadvertently contributed to the increasing rate of dieting and eating disorders in the population. This is an under-researched area, requiring further exploration.

The eating disorder field has accepted the calories-in, calories-out hypothesis without any criticism. The various forms of psychological treatments do not discuss the details of the diet, except for the need for regular eating and for weight restoration for malnourished patients.^38^^,^^97^ Hospital refeeding programs follow national dietary guidelines, and in the National Health Service tend to rely on processed foods with long shelf life for practical reasons.

Yet, at the same time, eating disorder clinicians have long described that patients binge on certain foods, which tend to be ultra-processed and high in both sugar and fats. The avoidance of these foods is not normally recommended treatment, although this area is starting to be explored.^98^^,^^99^

The main problem with the calories-in, calories-out hypothesis is that it doesn’t take into account the impact of various foods on appetite, satiety, and metabolism. Human beings are not burning fuel like a bomb calorimeter: humans have a digestive system with complex metabolic and neurobiological pathways that have developed over millions of years of evolution. Although the details are still not fully understood, the eating disorder field needs to pay attention to the metabolic and neurobiological effects of modern foods in the light of new research.

## CARBOHYDRATE-INSULIN MODEL

The carbohydrate-insulin model of obesity focuses on the endocrine and metabolic effects of food and has been garnering interest recently.^100^ According to this model, high-carbohydrate foods (various sugars and starches) are the most potent trigger of insulin, which is an important anabolic hormone. Its roles include stimulating glucose uptake into tissues, suppressing release of fatty acids from adipose tissue, inhibiting production of ketones in the liver, and promoting fat and glycogen deposition. Increased insulin is associated with increased appetite and weight gain. This model explains how the recent increases in the consumption of ultra-processed foods, with their high-glycemic-load carbohydrates, result in hormonal changes that promote lipid deposition in adipose tissue and exacerbate hunger. Although it continues to be an area of debate,^101^ recent animal and genetic studies suggest that insulin dysregulation plays a major role in obesity and metabolic disorders.^57^^,^^102^

Insulin is not just a peripheral hormone: it also acts on every cell in the body,^103^ including the brain.^104^ Its role in appetite and disordered eating was confirmed in a recent study of young people with type 1 diabetes, which found that bulimic symptoms were mainly physiologically driven by disrupted appetite regulation pathways resulting in uncontrollable hunger rather than psychopathology.^105^ These findings provide additional evidence that the treatment of these disorders should consider the metabolic and neuronal responses to foods consumed.

Current dietary guidelines recommend 45%–65% of energy intake from carbohydrates. However, historically, the range of energy intake from carbohydrates in the human diet was much lower than this. Access to high sugary foods in traditional societies was limited to fruit or honey, which were seasonal^106^ in most latitudes.^107^ The consumption of refined sugars only became widespread from the 19th century onwards; this resulted in increased rates of dental caries^108^^,^^109^ and obesity, as well as the start of the first cases of eating disorders.

Sucrose is a disaccharide that is broken down after ingestion to glucose and fructose, which have different metabolic and neurological pathways.^110^^,^^111^ Functional magnetic resonance imaging of the brain of healthy volunteers revealed that different simple sugars have a different impact on the brain, potentially mediated by differing insulin response. For example, fructose increases brain reactivity to food cues in the visual cortex more than glucose, paralleling the observation that fructose stimulates greater hunger and desire for food,^111^^,^^112^ whilst glucose is more satiating. High-fructose corn syrup is available in 45% glucose and 55% fructose, or 90% fructose, syrup, in the form of monosaccharides, which are metabolized more rapidly.

Furthermore, natural sources of carbohydrates exert different metabolic effects from processed foods. Starchy grains and vegetables contain polysaccharides, which break down to glucose in the body. The speed of this metabolism is affected by the fiber and fat content. Until recently, fructose was only available in the human diet for limited periods of time, usually in the autumn. Low doses of fructose are mainly cleared by the intestine, but high doses of fructose overwhelm intestinal fructose absorption, resulting in fructose reaching both the liver and colonic microbiota, which in turn initiates the processes leading to metabolic disorders such as fatty liver disease and insulin resistance.^102^ Johnson et al^113^ argued that the latter mechanism has an evolutionary advantage by increasing fat storage and allowing survival of food shortages in the winter. The permanent high-fructose consumption of NOVA-4 foods in the modern diet overrides this ancient mechanism and results in weight gain and metabolic disorders.^106^^,^^114^

Recent research using continuous glucose monitoring (CGM) has shown that glucose dysregulation is more prevalent and heterogeneous in the population than was previously thought and can affect individuals considered normoglycemic by standard measures.^115^ Zeevi et al^116^ found large individual differences in insulin and glucose responses to common foods, which are influenced by a number of factors, including sleep, stress, exercise, and the microbiome. They also found that the microbiome is influenced by environmental rather than genetic factors, and this includes the food environment.^117^ These findings may explain why some people develop certain metabolic or eating disorders in the modern food environment, whilst others do not.

For example, a systematic review and meta-analysis found that AN is associated with increased insulin sensitivity whilst BN and BED are associated with insulin resistance,^118^ and this is also consistent with genetic^24^ and epidemiological data regarding comorbidities.^119^^,^^120^ This variation in insulin sensitivity may contribute to the different responses to ultra-processed foods: people with BN and BED may be more prone to respond with high postprandial glucose spikes that drive hunger and appetite and fat accumulation, whilst patients with AN may have a blunted response and hence can tolerate hunger more. This possibility is supported by recent genetic research that found a negative association between type 2 diabetes and AN.^24^ The wider availability of CGM technology is likely to accelerate research in this area in the near future.

## NEW INGREDIENTS IN ULTRA-PROCESSED FOODS AFFECTING METABOLIC HEALTH

Ultra-processing is much more than just increased sugar content: it has also changed other components of widely consumed foods and has resulted in increased intake of substances that are not found in, or only exist in, small quantities in nature.

Animal fats have been replaced by industrial vegetable oils and trans fats, which have been shown to be pro-inflammatory and to impair appetite regulation.^121^^,^^122^ Vegetable oils have been promoted as healthier alternatives to saturated fats, as they can reduce cholesterol levels; consequently, their consumption has dramatically increased worldwide.^123^ Vegetable oils are high in omega-6 polyunsaturated fatty acids, the intake of which was historically low in the human diet.^124^ Until the turn of the 20th century, the ratio of omega-6:omega-3 intake was 1:1, and this has increased to 20:1 in the Western diet in parallel with the obesity epidemic.^125^ Experimental work shows that linoleic acid is toxic to beneficial bacteria in the gut,^126^ which may explain why the increased ratio of omega-6 to omega-3 has been linked with inflammation and metabolic disorders as well as depression and eating disorders.^125^^,^^127^^,^^128^ Disappointingly, interventions aimed at improving omega-6:omega-3 ratios by using fish oil supplements have yielded conflicting results.^129^^,^^130^ This could have been due to the fact that the addition of supplements was not sufficient to reverse the metabolic effects of the patient’s background diet. Future research should focus on the effects of reintroducing minimally processed (NOVA 1–2) foods in relation to treating individuals with eating disorders and improving omega-6:omega-3 ratios.

Food manufacturing has also resulted in a steady increase in consumption of food additives.^73^ Currently, 1400 molecules are approved as food additives by the European Union (https://www.food.gov.uk/business-guidance/eu-approved-additives-and-e-numbers). These did not exist in the human diet before the introduction of NOVA-4 foods. The metabolic consequences of these additives are poorly understood.

For example, a recent study has shown that propionate, a widely used food additive to prevent molding in baked goods, reconstituted meat, and dairy products, leads to insulin resistance and hyperinsulinemia, both in animals and humans.^131^

Emulsifiers and enzymes such as transglutaminase are added to extend shelf life or improve the consistency, texture, and palatability of foods in a range of products, such as low-fat dairy, reconstituted meat, and cereals.^132^^,^^133^ Animal experiments have shown that emulsifiers damage the mucus structure that protects the intestinal wall and trigger inflammation and metabolic syndrome/obesity through changing the microbiota.^134–136^ This is relevant to patients with eating disorders, who often use low-fat products in an attempt to control their calorie intake and often report abdominal complaints, such as irritable bowel syndrome.^136^ Transglutaminases are also associated with autoimmune disorders.^137^ This needs to be further explored, as recent studies have shown an increased rate of autoimmune disorders among those with eating disorders.^23^^,^^54^^,^^138^

Artificial sweeteners have been introduced into modern foods since the 1980s. They have been heavily promoted by the diet industry and endorsed by medical bodies as “healthier” alternatives to sugar to help prevent weight gain and manage diabetes.^139^^,^^140^ A recent US study showed 25.1% of children and 41.4% adults consumed these products – 80% on a daily basis.^141^ They are commonly used in diet products, sometimes in combination with nutritive sugars, without the consumers being aware of their inclusion. Whilst they are noncaloric, there is increasing controversy regarding their potential to promote metabolic abnormalities in humans.^142^ It has been demonstrated that they induce glucose intolerance both in animal experiments and in humans, by functionally altering the gut microbiome.^143^^,^^144^ Furthermore, they also affect multiple brain pathways, including those relating to sleep and appetite.^145^ Patients with eating disorders often consume large amounts of diet drinks, which suggests an increased desire for sweet taste, but this may have negative metabolic consequences, which further drives disordered eating.^146–148^ To our knowledge, the role of artificial sweeteners in maintaining the binge–purging cycle has not been studied. Given the higher use of artificial sweeteners by patients with eating disorders, there is a need for further research in this area.

The biological and metabolic effects of ultra-processed foods have been studied using the “cafeteria diet” in animal experiments. In this model, researchers replace standard chow with human cafeteria foods (eg, cookies, cereals, cheese, processed meats, crackers – all high in sugars, vegetable oils, and additives). Animals fed on these foods exhibit voluntary hyperphagia, which results in dramatic weight gain. Furthermore, cafeteria diet feeding promotes a prediabetic condition, with elevated glucose, insulin, and nonesterified fatty acids, accompanied by decreased insulin tolerance. In addition, chronically inflamed liver and adipose tissues and distorted pancreatic islet architecture develop. In contrast, animals fed a high-fat, lard-based diet with a similar level of fat content (45%) do not develop these changes, as they reduce their food intake and maintain their weight, whilst this autoregulatory mechanism is impaired in the cafeteria diet group.^149^^,^^150^ Furthermore, cafeteria diet has a profound impact on the gut microbiome, which, in turn, may be associated with important features of metabolic syndrome.^151^ The impact of ingredients in the modern diet that can cause dysbiosis needs to be further investigated given that the gut microbiota not only influences host metabolism but can also affect brain function and behavior through the microbiota-gut-brain axis.^152^^,^^153^ This remains an under-researched area, which will need more attention in the future.

This experimental observation seems to mirror the human experience: since the introduction of ultra-processed foods, the majority of the population overeats. Furthermore, the same increase in low-grade inflammation is seen in human metabolic disorders and BED.^154^ A recent, carefully controlled human trial confirmed that participants consumed 500 kcal/d in excess when they were placed on an ad libitum, ultra-processed diet, as compared with a minimally processed one, with profound changes in metabolic parameters.^155^ The appetite-suppressing hormone peptide tyrosine tyrosine increased when participants followed the unprocessed diet as compared with both the ultra-processed diet and baseline. In contrast, levels of the hunger hormone ghrelin, along with fasting glucose and insulin levels, were increased during the ultra-processed diet. Interestingly, only protein intake levels were stable between the two groups: ultra-processed diet increased both carbohydrate and fat intake. This is an important experimental study, demonstrating that overeating on ultra-processed foods is biologically driven not just in animals but also in humans.

## EFFECTS OF ULTRA-PROCESSED FOODS ON THE BRAIN

There has been much research on the neurobiology of eating disorders, including the brain’s reward systems as well as the response to different foods.^156^ Food addiction as a concept emerged in 2010, when the first studies showed that cafeteria diet causes alterations in the dopaminergic reward systems; this concept has been gaining increasing popularity among eating disorders researchers.^157^^,^^158^ However, it remains controversial: whilst there may be several components in foods that impact on central dopamine receptors, the term *food addiction* implies that any food can be addictive. Again, sugar emerges as a main potentially addictive substance that acts on multiple pathways.^159^ Food addiction also implies that it is the individual’s choice or fault. However, there are some fundamental differences between addiction to food versus other substances: whilst substance misuse is the individual’s choice, eating is necessary for life. The potentially metabolically harmful ingredients, such as high sugar content and additives, in ultra-processed foods have been added without informing the consumers.^160^ Some of these effects are known (as discussed above), but others are yet to be discovered.

Small and DiFeliceantonio^161^ have been working on elucidating the neurobiological pathways affected by processed foods. In functional magnetic resonance imaging studies they have established that food cues, which are predictive of calories, activate the striatum in humans; that the magnitude of these responses is regulated by metabolic signals independently of liking; and that this process regulates food intake according to energy requirement.^162^ Under normal circumstances, this mechanism prevents over- or undereating. Nonnutritive sweeteners, which are not found in nature, and combinations of sugars and fats, can override the metabolic signals, resulting in supra-additive effects.^163^ Interestingly, individuals are better able to estimate the energy density of high-fat and high-protein foods (such as cheese or meat products) vs carbohydrate only (sweets), or carbohydrate combined with fat (cakes, biscuits, etc.). Also, the use of noncaloric sweeteners disrupts the brain’s ability to accurately estimate the energy value of foods.^161^ This effect is associated with functional connectivity between visual (fusiform gyrus) and valuation (ventromedial prefrontal cortex) areas. The findings are consistent with evidence from ecological studies and randomized controlled trial showing overconsumption of ultra-processed foods.^73^^,^^155^

Although these studies were carried out using only a small range of foods, these discoveries are highly relevant to understanding the pathomechanisms of eating disorders: patients typically binge on ultra-processed foods with high sugar-fat combinations, with a baseline of using diet products.

Apart from neurobiology and the microbiome, many neuroendocrine pathways also affect dietary intake.^104^ Discussing all potential factors is beyond the scope of this review. Among others, stress hormones are highly important, as they influence food preference and consumption, acting on reward circuitry. Acute stress suppresses appetite in people of normal weight, whilst chronic stress and high cortisol levels increase selection of high-calorie, palatable foods. In contrast, in overweight or obese individuals, acute stress may influence brain response to promote eating.^164^ This is consistent with the observation that people with AN reduce their dietary intake under stress, whilst people with BN and BEDs increase their intake of palatable foods. The metabolic effects of ultra-processed foods further drive this vicious cycle. Consumption of glucose, sucrose, or fructose results in caloric overconsumption through activation of ghrelin and depressed satiety signals.^165^

Many other important hormones regulate satiety. For example, cholecystokinin and plasma peptide tyrosine tyrosine are important satiating hormones, which mainly respond to protein and fat consumption.^166^^,^^167^ The roles of fat and protein intake have been less explored in the context of appetite regulation,^168^^,^^169^ and further work is needed on this topic. This has relevance to the treatment of eating disorders, as it has been described that protein intake is low in this patient population.^170^

Small and DiFeliceantonio^161^ have developed a 2-stage model bringing together the biological and psychosocial factors influencing eating behaviors: one system directly reflects the nutritional value of foods and relies on metabolic signals reaching the brain. This nutrient-sensing system appears to play a critical role in regulating striatal dopamine, determining the value of foods, and driving food choice. In the second system, conscious perceptions such as flavor and beliefs about caloric content, cost, and healthfulness of foods are also important determinants of food choice. The latter is dependent on circuits within the prefrontal cortex and insular cortex. This model significantly advances our understanding of the pathomechanisms involved in eating disorders.

## IMPLICATIONS FOR THE ETIOLOGY AND TREATMENT OF EATING DISORDERS

The present summary of the recent literature brings together a number of different strands of research that have so far been considered in isolation, but which when brought together provide a more complete understanding of biological and psychosocial pathomechanisms involved in eating disorders. This can potentially fill the gaps in understanding the biological processes that are at play, and can provide new options for improving treatment effectiveness.

**Figure 5:**
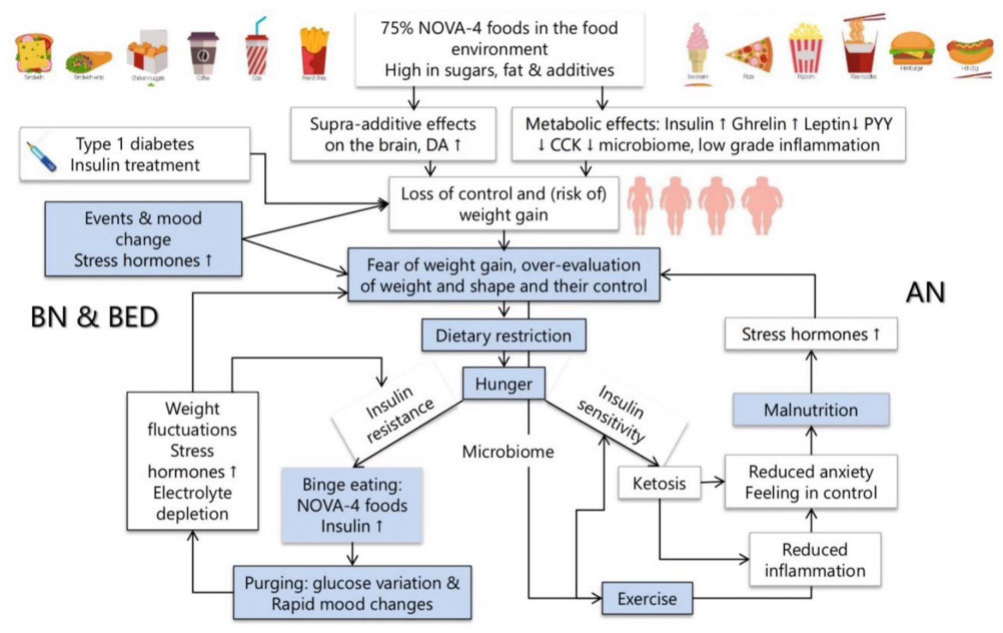
Integration of the psychological, metabolic, and neurobiological maintaining factors involved in eating disorders. *Abbreviations:* AN, anorexia nervosa; BED, binge eating disorder; BN, bulimia nervosa; CCK, cholecystokinin; DA1, dopamine D1 receptor; PYY, peptide tyrosine tyrosine

Existing theoretical models focus on individual psychopathology or family factors, but there has been almost no exploration of the metabolic and neurobiological responses to foods. The lack of awareness by clinicians of these has led to a standard approach to encourage all patients to widen their food choices regardless of their potential individual metabolic consequences. A paradigm shift is needed in the conceptual framework by which we understand the vulnerability to and the maintenance of different eating disorders, by integrating recent knowledge of the biological impact of modern highly processed foods, and individual metabolic differences into existing psychological models. Such a model is outlined in Figure 5, building on Fairburn’s transdiagnostic model.^13^ This new integrated model could improve treatment outcomes and reduce the personal and societal costs of these illnesses. The various forms of eating disorders may share psychopathology, but they are likely to have different metabolic pathways, depending on individual biological variations, such as insulin sensitivity, dietary choices and microbiome.^117^^,^^171^^,^^172^

The central feature of psychopathology is the overevaluation of, and control of, weight and shape.^13^ Patients with eating disorders commonly fear spiralling out of control and being unable to stop eating. This is a real risk in the modern food environment, where high-carbohydrate, ultra-processed foods are ubiquitous. As the previous sections outlined, these foods promote overeating by impairing multiple endocrine and neurobiological mechanisms. This increases the risk of becoming overweight or obese, which in turn increases the fear of weight gain. One can argue that people with eating disorders try to replace an automatic mechanism to ensure the maintenance of normal weight by a conscious effort, following the health messages in the environment, which encourage fat intake reduction, calorie counting, and exercise. Unfortunately, none of these strategies are effective, and they continue to drive disordered eating and preoccupations.

The effectiveness of psychological treatment could be much improved by including a personalized plan that eliminates ultra-processed foods and reduces sugar and carbohydrate, while increasing high-fat, moderate-protein natural foods. This approach would offer the patient a sustainable solution to maintaining an optimal weight without the risk of weight gain. It could also help challenge fat phobia and reduce abdominal symptoms. Such a dietary change may also help improve comorbid depression, which is very common in eating disorders.^173^^,^^174^ The risk of increasing the patient’s preoccupation with new dietary rules can be managed in a variety of ways. These include psychoeducation and encouragement of freshly prepared, home-cooked family meals, which have been shown to be protective of both eating disorders and obesity.^175^^,^^176^ Furthermore, modern technology, such as CGM, allows for immediate and personalized feedback to the patient about the metabolic effects of foods, which can be incorporated into psychological treatment or even used for remote monitoring. Such models have been successful in facilitating lifestyle changes in other high-risk patient groups, such as in those with diabetes.^177^

The subsequent sections discuss the implications on different types of eating disorders, depending on their respective metabolic vulnerability.

### Eating disorders in diabetes

The co-occurrence of diabetes and eating disorders suggests potential shared pathomechanisms. Insulin is the most likely common denominator: its involvement in diabetes is well known and its impact on satiety and eating behavior has been discussed earlier. One recent study found that 98% of individuals with type 1 diabetes engage in disinhibited eating during perceived episodes of hypoglycemia, which is related to a mismatch of the dose of insulin and dietary carbohydrate intake, and up to 50% develop disturbed eating behaviors after the diagnosis and commencing insulin treatment.^178^

A number of models have been proposed for the maintenance of eating disorders in type 1 diabetes, which consider the biological and metabolic effects of insulin and disruption of hunger and satiety regulation in addition to traditional psychological factors.^179^^,^^180^ None, however, discuss the impact of food choices.

Research has shown that people with diabetes and eating disorders do not respond to standard eating disorder treatment and have high dropout rates from psychological therapies.^179^ This suggests that patients find the current mainstream treatment, which encourages continuing moderate carbohydrate intake with matched insulin treatment, unhelpful in preventing further weight gain. After initiating insulin treatment, patients gain weight, which contributes to fear of further weight gain particularly in young women, with consequent disordered eating and insulin omissions, which can be highly dangerous. Furthermore, patients are often exposed to conflicting messages from the two disciplines: eating disorder specialists actively discourage restriction of any type of foods, and diabetic teams recommend calorie and carbohydrate counting, weight loss, and use of low-fat products. The lack of an integrated approach may explain poor response rates and disengagement and inadvertently reinforces eating disorder psychopathology.

The link between type 2 diabetes and eating disorders is bidirectional. People with BN or BEDs have a higher risk of type 2 diabetes during their lifetime, which may be related to hyperinsulinemia, which often exists decades before the diagnosis and can be aggravated by ultra-processed foods, which are high in sugars and metabolically active additives.^181^^,^^182^

Interestingly, there has been very little discussion of the potential benefits of reducing carbohydrate intake in the management of eating disorders, either with or without comorbid diabetes. Recent research has shown that ultra-processed foods, which combine high levels of carbohydrates and fats, have a supra-additive effect on the brain.^161^ Recent studies have shown that a high-fat, low-carbohydrate (HFLC) diet can be helpful in both type 1 and type 2 diabetes and can improve various metabolic parameters, including normalization of weight without the need to calorie count.^177^^,^^183^^,^^184^ The use of carbohydrate restriction is not new in the treatment of diabetes, but was almost entirely forgotten until the most recent inclusion in US and UK guidelines.^185^

Although more research is needed, an increasing number of patients have had success adopting a low-carbohydrate lifestyle, which is now supported by various high-quality online resources. For example, the Low Carb Program (https://www.lowcarbprogram.com/) is an award-winning resource in the UK for individuals with metabolic disorders, which has 425 000 subscribers at the time of writing and can now be prescribed by the National Health Service. It has been shown to be effective in helping people with type 2 diabetes to achieve better glycemic control, weight loss, and a reduction in hypoglycemic medications, offering significant cost savings to the National Health Service.^186^

Less research has been conducted on type 1 diabetes, but initial results are encouraging.^187^ A recent online survey of children and adults with type 1 diabetes who adopted a very-low-carbohydrate lifestyle (meaning 36 g/d) reported exceptional glycemic control with low rates of adverse events.^183^

Taken together, the evidence strongly suggests that adopting a HFLC diet would be beneficial for patients with diabetes and comorbid eating disorders for the following reasons: It would help achieve stable glucose control, reduce the need for insulin, stabilize satiety and appetite, and reduce risk of complications (both in terms of weight gain and microvascular damage). It would also help to reduce the glucose fluctuations related to sporting activities, which would have additional physical and mental health benefits. Further studies are urgently needed in this patient population.

### Bulimia nervosa and binge eating disorder

As explained above, current psychological therapies focus on the psychological and behavioral aspects of these disorders, and there is limited exploration of the underlying pathophysiology. Little attention is given to the diet itself, and avoidance of any specific foods is actively discouraged. Furthermore, weight loss is not addressed in the treatment of BED, which is a significant limitation for these patients, whose physical health is often compromised by comorbid obesity and who would like to lose weight.

As discussed, people with BN and BED have increased rates of insulin resistance and metabolic disorders, are more likely to be overweight or obese, and have increased risk of developing diabetes.^118^ Recent studies have shown that there is high variability in an individual’s glucose responses to common foods, even in nondiabetic populations,^115^^,^^116^ and this is likely to be relevant to people with BN and BED, although no studies have yet been conducted in this patient population. It is possible that a combination of an individual’s metabolic vulnerability (eg, hyperinsulinemia) triggers a cascade of events when consuming ultra-processed foods that are high in sugars. These may include rapid changes in blood glucose levels, resulting in excessive appetite, which is further derailed when using binge foods that combine high sugar and fat content. We are not aware of any studies of subjects with BN and BED that use CGM to monitor their blood glucose response to foods. This new technology provides a tool for further research that can transform the field in the coming years.

Current psychological treatments propose regular eating (4–6 times a day) as an essential component of treatment to reduce bingeing and purging.^38^ Instead, it is likely that if the patient continues consuming ultra-processed foods (which is usually the case), the risk of ongoing hunger, weight gain, and further deterioration of metabolic parameters is increased, which in turn drives the psychopathology. Psychological treatment should integrate the biological consequences of the patient’s diet. A personalized approach can individually determine the optimal diet composition based on glucose and insulin monitoring, as shown by Zeevi et al.^116^ The removal of ultra-processed foods and reduction of carbohydrate intake to a low level, replacing them with unprocessed and minimally processed foods high in fat and protein, is likely to be beneficial for these patients by improving satiety and reducing food cravings.^188^

Although we are not aware of HFLC trials in BN or BEDs, one recent randomized controlled trial of a very-low-calorie ketogenic diet in obese men and women showed significant improvements, not just in weight but also in food-craving scores and overall quality of life.^189^ Similar results were reported by another group using ketogenic diet.^188^ This approach could provide a new alternative for the treatment of people with BED who wish to lose weight, which is not addressed in current psychological treatments. Technological advances, such as CGM, can provide real-time personalized information as to the impact of the diet on the patient’s own biology, and this can be integrated into psychological treatment. Furthermore, exercise also has a positive effect on insulin sensitivity, which can help to stabilize appetite. One novel randomized controlled trial compared cognitive-behavioral therapy with a combination of exercise and dietary advice for patients with BN and BED^190^ and reported faster improvement in the diet and exercise group at the Academy for Eating Disorders conference in 2019. Future work is needed to establish the optimal diet and exercise approach for the treatment of BN and BED that would enhance psychological treatments.

### Anorexia nervosa

Patients with AN severely restrict their diet to achieve weight loss and then develop protein-energy malnutrition. Weight restoration is an essential part of treatment,^10^ but there has been no work done on comparing the effectiveness of refeeding regimes using different types of foods or macronutrients. It is well known that patients report a sense of well-being and reduced anxiety during starvation, which is lost with weight restoration. This is one of the reasons why many resist refeeding even though they may understand that it is necessary for recovery. Scolnick^191^ has raised the possibility that this sense of well-being is likely to be related to nutritional ketosis, which is a metabolic response to fasting. Ketosis is an evolutionarily conserved mechanism that is an ancient adaptation to periods of food shortages, in particular reduced carbohydrate intake. This leads to a fundamental biochemical change in the metabolic supply of the brain and muscle, in which glucose is partially replaced with ketone bodies, which are produced by the liver and by certain microbes in the colon. Beta-hydroxybutyrate is an important metabolite that can be used by the brain, heart, and muscle as alternative fuel to glucose.^192^ It is also an important signaling molecule, with anti-inflammatory and anxiolytic effects.^193–195^ This may explain why patients with severe anorexia nervosa can have asymptomatic hypoglycemia, which has puzzled clinicians. Interestingly, although the presence of ketosis in starvation and in patients with AN is well known to clinicians, no research has explored its significance. It would be important to investigate this further. The impact of ketone bodies on the brain has been known since the 1920s, when their powerful antiepileptic effect was discovered. Since then, ketogenic diet has been used in treatment-resistant epilepsy, particularly in children, with good effects.^196^ A recent study has demonstrated positive effects of nutritional ketosis on mood and quality of life,^189^ and there is increasing research interest in this area.^174^^,^^195^

While preparing this article, no studies were found that compared processed and unprocessed foods, or high- or low-fat diets, or diets with varying protein content (despite the protein-energy malnutrition in AN), yet such studies are urgently needed given that relapse rates after hospital refeeding programs are high.

There are a number of unpublished case reports of patients with AN who benefited from an HFLC diet, as reported by Scolnick^191^ and Unwin, written personal communication. Although it may sound counterintuitive to use HFLC diet for weight restoration, there are a number of potential advantages of an HFLC diet in this patient population. These include the reduced risk of refeeding syndrome (which occurs as a result of switching from fat to carbohydrate metabolism) and the maintenance of nutritional ketosis and associated well-being due to the anxiolytic effects of beta-hydroxybutyrate without the harms of malnutrition. It would also challenge the fear of fats early in treatment. Furthermore, an HFLC diet uses unprocessed foods and is satiating and therefore less likely to trigger transition to BN and binge eating, which is a risk in 20%–30% of patients, and may alleviate the commonly experienced concerns about losing control of eating. Such diets prevent hyperinsulinemia, which is the mechanism underlying abdominal fat accumulation. Standard dietary approaches to weight restoration, which often include processed foods with high sugar content, often result in central fat accumulation. For patients with AN this is a highly distressing experience that can worsen preoccupations with weight and shape, and the desire to lose weight, and may result in poor compliance with treatment and a high rate of relapse.^197^

Further research – to compare the response to a HFLC diet using minimally processed foods with treatment as usual – is urgently needed. Our own clinical experience is that patients with AN are open to the introduction of high-fat, HFLC foods after they understand the underlying theory and potential benefits. However, they need intensive support to implement changes in the correct manner, both at the beginning of treatment when weight gain is required and until maintenance is achieved. The elimination of ultra-processed and diet foods could help restore normal satiety mechanisms and metabolic health, but it may not be culturally easy given the ubiquitous presence of these foods. However, this is in line with the World Health Organization’s call for a reduction in NOVA-4 foods: “the ever-increasing production and consumption of these products is a world crisis, to be confronted, checked and reversed as part of the work of the UN Sustainable Development Goals and its Decade of Nutrition.”^73^

For an HFLC ketogenic diet to be successful in the treatment of AN, careful consideration is necessary. It needs be designed to meet increased energy and protein requirements. Remote monitoring and support are new technologies that can be helpful in achieving lasting behavioral change, and this approach can be fully integrated into psychological treatment. Although there are plenty of publicly available online resources to help patients and families introduce a high-fat HFLC ketogenic diet by using natural foods, the majority of these focus on weight loss and, hence, are not appropriate for individuals with AN, who need to gain weight. The common risk associated with a HFLC diet can be safely managed. These include hyponatremia, which can be easily prevented by ensuring adequate salt intake.

## CONCLUSION

In summary, recent epidemiological trends highlight that disordered eating is associated with the obesity epidemic and the parallel increase in metabolic disorders. Over the last 50 years, there has been a fundamental change in the food environment: ultra-processed foods have been increasingly replacing unprocessed seasonal foods. This change has not been metabolically inert: the increased consumption of various sugars drives hyperinsulinemia and insulin resistance, and the metabolic consequences of the large number of food additives are only now beginning to emerge. Many of these are associated with low-grade inflammation, microbiome changes, and impairment of intestinal permeability, along with an increase in autoimmune diseases. Furthermore, recent experimental data have also shown that highly processed foods can lead to overconsumption by impairing multiple metabolic and neurobiological pathways. These findings are highly relevant to the development, maintenance, and treatment of eating disorders, particularly given that there is emerging evidence of insulin dysregulation in BN and BED and insulin sensitivity in AN.

Future research in the field should explore the underlying neurobiological and metabolic responses to food choices and integrate them with psychological treatment (Table 3). This should include an exploration of individual responses to food choices and their roles in the development and maintenance of the disorder. Randomized controlled trials are needed to compare the effects of diets with differing macronutrients and reduced NOVA-4 foods. This could provide new opportunities for prevention and improving treatment outcomes, which have been stagnating over the last few years.
